# Dysferlin-deficient immortalized human myoblasts and myotubes as a useful tool to study dysferlinopathy

**DOI:** 10.1371/currents.RRN1298

**Published:** 2012-02-28

**Authors:** Susanne Philippi, Anne Bigot, Andreas Marg, Vincent Mouly, Simone Spuler, Ute Zacharias

**Affiliations:** ^*^Muscle Research Unit, ECRC, MDC, Berlin, Germany; Université Pierre et Marie Curie UM76/INSERM U974, CNRS UMR 7215, Institut de Myologie, Groupe Hospitalier Pitié-Salpetrière, Paris, France; ^†^Université Pierre et Marie Curie UM76/INSERM U974, CNRS UMR 7215, Institut de Myologie, Groupe Hospitalier Pitié-Salpetrière, Paris, France; ^‡^Muscle Research Unit, Experimental and Clinical Research Center, a joint cooperation between the Charité Medical Faculty and the Max-Delbrück Center for Molecular Medicine, Berlin, Germany and ^§^DR2 in Université Pierre et Marie Curie UM76/INSERM U974, CNRS UMR 7215, Institut de Myologie, Groupe Hospitalier Pitié-Salpetrière, Paris, France

## Abstract

Dysferlin gene mutations causing LGMD2B are associated with defects in muscle membrane repair. Four stable cell lines have been established from primary human dysferlin-deficient myoblasts harbouring different mutations in the dysferlin gene. We have compared immortalized human myoblasts and myotubes carrying disease-causing mutations in dysferlin to their wild-type counterparts. Fusion of myoblasts into myotubes and expression of muscle-specific differentiation markers were investigated with special emphasis on dysferlin protein expression, subcellular localization and function in membrane repair. We found that the immortalized myoblasts and myotubes were virtually indistinguishable from their parental cell line for all of the criteria we investigated. They therefore will provide a very useful tool to further investigate dysferlin function and pathophysiology as well as to test therapeutic strategies at the cellular level.

## Introduction  

Muscular dystrophies comprise clinically and genetically heterogeneous disorders characterized by progressive weakness and wasting of the skeletal muscle accompanied by an increase in muscle connective tissue [1] Dysferlin gene mutations cause limb girdle muscular dystrophy 2B (LGMD2B) and Miyoshi myopathy, allelic autosomal recessive diseases characterized by limb girdle or distal weakness of early adult onset  [2] [3]. Dysferlin (MIM*603009) is a 230kDa transmembrane protein comprising calcium binding C2 domains that is highly expressed in skeletal muscle [4] [5]. Dysferlin localizes to the sarcolemma and is involved in membrane repair, membrane trafficking and muscle regeneration [6] [7] [8]. Various mutations associated with LGMD2B have been identified in dysferlin. These mutations lead either to a reduced expression of dysferlin at the sarcolemma, an intracelluar accumulation of dysferlin, the formation of amyloid-like deposits or to the complete absence of dysferlin protein finally resulting in impaired muscle membrane repair [9] [10] [11].

The access to primary human myoblasts from biopsies of patients with disease-causing dysferlin mutations is limited. Due to excessive fibrosis, these muscle biopsies often contain only very few myogenic cells and are highly intermingled with connective tissue cells like fibroblasts and adipocytes. Additionally, primary human myoblasts in culture show a limited proliferative potential and undergo changes that are linked to replicative senescence [12]. To circumvent these limitations immortalized human myoblast lines were generated by retroviral transduction of primary human myoblasts harbouring different disease-causing mutations with telomerase (hTERT) and cyclin-dependent kinase 4 (CDK-4). The expression of hTERT overcomes the progressive erosion of telomeres occuring due to cell division and the overexpression of CDK-4 blocks the induction of the p16-mediated cellular stress-pathway [13]. After their immortalization these cell lines show a prolonged proliferation and differentiation capacity compared to primary human myoblasts *in vitro* and they can be transplanted into regenerating muscle *in vivo* [13].

Here we describe a detailed analysis of immortalized human myoblast lines harbouring different dysferlin mutations. These cell lines maintain the characteristics of primary dysferlin-deficient human cell strains with respect to myogenic differentiation, dysferlin expression and membrane repair and represent a useful tool to further investigate all aspects of dysferlin function and dysfunction. 

## Material and Methods

### Patients

The Charité internal review board approved the study and written informed consent was obtained from all patients. Skeletal muscle biopsies (M. vastus lateralis) were obtained from four patients with dysferlinopathy and four healthy controls. Patients with LGMD2B were affected by the following mutations in dysferlin: homozygous c.4022T>C (DYSF1), compound heterozygous c.855+1delG/c.895G>A (DYSF2), compound heterozygous c.1448C>A/c.*107T>A (DYSF3) and homozygous c.2810+2T>A (DYSF4) (see Table1) resulting either in complete loss or intracellular aggregation of dysferlin. At the time of biopsies taken patients were 57, 37, 36 and 25 years old, respectively.  

### Purification and differentiation of primary human myoblasts

Primary myoblasts were isolated by protease digestion from fresh muscle biopsies and expanded at 37^o^C in humidified atmosphere at 5% CO_2_ in skeletal muscle growth medium (PromoCell, Heidelberg, Germany) supplemented with 10% FCS, glutamine (3mM) and gentamycin (40µg/ml) (Gibco, Paisely, UK). All cultures were enriched in myoblasts by immuno-magnetic cell sorting using anti-CD56/NCAM antibody coated magnetic beads (Miltenyi Biotech, Bergisch Gladbach, Germany). Purity of the myoblast preparation was verified by staining with an anti-desmin antibody (DAKO) revealing more than 95% desmin-positive cells. Differentiation of myoblasts into myotubes was initiated at approximately 90% confluence by cultivation in differentiation medium (DMEM, 2% horse serum) for 7 days.  

### Immortalization of primary human myoblasts and their differentiation into myotubes

Primary human dysferlin-deficient and control myoblast lines were transduced with pBABE retroviral vectors carrying Cdk4 and hTERT. Puromycin and neomycin were used as selection markers, respectively and isolation of individual myogenic clones was carried out as described by Mamchaoui et al. [13]. The immortalized dysferlin-deficient and control human myoblast lines were cultured in growth medium consisting of 1 vol 199 Medium (Invitrogen, Carlsbad, CA)/4 vol DMEM (Invitrogen) supplemented with 20% foetal calf serum (Invitrogen), 2.5 ng/ml HGF (Invitrogen), 0.1 µM Dexamethasone (Sigma-Aldrich, St. Louis, MO) and 50µg/ml Gentamycin (Invitrogen). Differentiation into myotubes was initiated at approximately 90% confluence by cultivation in differentiation medium (DMEM, 2% horse serum) for 7 days. 

### Immunocytochemistry

Myotubes were fixed for 10 min with 4% formaldehyde, permeabilized for 15 min with 0.2%Triton X100 and blocked with 1% BSA diluted in PBS. The following primary antibodies were used for immunostaining: mouse monoclonal antibodies to MHCs and dysferlin HAMLET (both from Novocastra, Newcastle upon Tyne, UK), desmin (Dako), caveolin3 (Santa Cruz Biotechnology), MHCf  and a-actinin (both from Sigma-Aldrich) in combination with secondary Alexa 488- or Alexa 568-conjugated anti-mouse IgG antibodies (Invitrogen). Nuclei were counterstained with Hoechst 33258. Images were collected using a Leica DMI6000 fluorescence microscope or with a Zeiss-LSM 510 META confocal microscope. 

### SDS-PAGE and Western blot analysis 

Myotubes were lysed and homogenized in protein extraction buffer (50mM Tris pH 7.4, 150mM NaCl, 0.5% Triton X-100, 0.5% Na-deoxycholate,1mM EDTA, 50mM NaF, 1mM Na-vanadate including protease inhibitors [Pierce]). Total protein extracts (15µg) were separated on 10% SDS PAGE and transferred onto nitrocellulose membranes. Membranes were blocked in 5% skimmed milk in TBS-tween 20 (0.05%) and probed with one of the following antibodies: mouse monoclonal antibodies to MHC (MF20; DSHB, Iowa City, IA,USA), desmin (Dako), dysferlin (HAMLET, Novocastra, Newcastle upon Tyne), a-tubulin (Sigma-Aldrich), caveolin3 (Santa Cruz Biotechnology) and a rabbit polyclonal antibody against dysferlin [10] in combination with secondary goat anti-mouse IgG or goat anti-rabbit IgG IRDye 800-conjugated antibodies (LI-CORE, Lincoln, Nebraska USA). Immunoblots were visualized using the Odyssey infrared imaging system (LI-CORE). 

### Laser mediated membrane wounding 

Shortly before performing the membrane wounding myotubes were washed once in Tyrode solution (140 mM NaCl, 5 mM KCl, 2 mM MgCl_2_, 2.5 mM CaCl_2 _and 10 mM HEPES, pH 7.2). The wounding experiment was performed in Tyrode solution supplemented with the FM1-43 dye (2.5µM; Molecular Probes, Invitrogen, Paisley, UK). Myotubes were wounded by the irradiation of a 2.5 x 2.5 μm surface area for 58 s at 50% of the laser power (30mW argon-laser) employing a Zeiss-LSM 510 META confocal microscope. Images were taken with a 63x oil immersion objective every 20s for 280s after wounding and were processed using the Zeiss LSM Image Browser software. Changes of fluorescence intensity were calculated using ImageJ.  

## Results 

### Generation of dysferlin-deficient immortalized human myoblast lines

Four stable cell lines were established from primary human dysferlin-deficient myoblasts harbouring different mutations in dysferlin resulting in either the intracellular aggregation of dysferlin (DYSF1 and DYSF2) or the complete absence of dysferlin protein (DYSF3 and DYSF4) as indicated in Table1.

After cloning, all immortalized myoblast lines were 100% myogenic as shown by the expression of both desmin and CD56/NCAM by immunocytochemistry, with a total absence of connective tissue cells such as fibroblasts or adipocytes. 

**Table1** Summary of myoblast lines harbouring different mutations in dysferlin  

**Table:** 

Myoblast line	Exon	State	Nucleotide change	mRNA change	Protein change
DYSF1	38	homozygous	c.4022T>C	r.4022u>c	p.L1341P
DYSF2	8i 9	heterozygous	c.855+1delG c.895G>A	splice site mutation r.895g>a	p.G299R
DYSF3	16 55	heterozygous	c.1448C>A c.*107T>A	r.1448c>a r.*107u>a	p.S483X
DYSF4	26i	homozygous	c.2810+2T>A	splice site mutation	

The reference sequence used is GenBank ID NM_003494.  The nomenclature sequence uses “c”, “r” and “p” when referring to cDNA, mRNA and protein, respectively, and “i” when referring to intronic sequence. 

### Comparison of dysferlin-deficient immortalized human myoblasts and myotubes to their parental cell lines 

The effect of immortalization on myoblast differentiation into myotubes was analyzed by Western blott assessing the expression of myogenic differentiation markers. All immortalized human myoblast lines showed a strong expression of desmin and a weak expression of caveolin3 (Fig.1B) similar to their parent myoblast lines (Fig.1A). Primary and immortalized (IM) wild-type human myoblasts showed a low expression of dysferlin, that was reduced in IM DYSF1 and DYSF2/IM DYSF2 and completely absent in DYSF3/IM DYSF3 and DYSF4/IM DYSF4. We were not able to analyze primary human DYSF1 myoblasts and their corresponding myotubes due to a massive presence of non-muscle cell types and a very poor myogenicity, further enhancing the interest in generating immortalized clones from patients’ primary cultures. 

After 7 days of differentiation myotube formation was associated with a strong increase in the expression of myosin heavy chain (MHC) and caveolin3 in all primary and immortalized cell lines investigated (Fig.1A and 1B) except DYSF1 regardless of the presence of dysferlin. Desmin was still expressed in myotubes. a-tubulin served as a loading control. Myotubes derived from primary and immortalized wild-type myoblasts showed a strong increase in dysferlin expression with differentiation (Fig.1A and 1B). As expected, myotubes with disease-causing mutations in DYSF showed a strongly reduced expression (IM DYSF1 and DYSF2/IM DYSF2) or a complete absence of dysferlin protein (DYSF3/IM DYSF3 and DYSF4/IM DYSF4).

**Figure:**
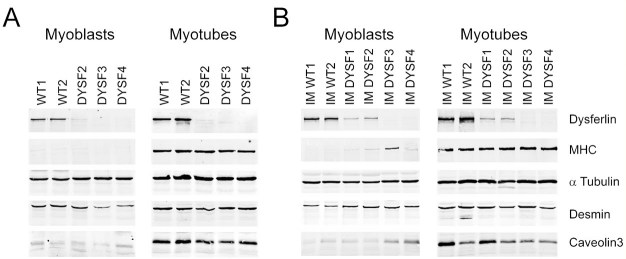
**Fig.1 **Comparison of immortalized human myoblasts and myotubes to their parental counterparts.The expression of myogenic differentiation markers was analysed by Western blot in dysferlin-deficient and wild-type human myoblasts and myotubes in primary cells (A) and after immortalization (B). A similar increase in MHC (200kDa) and caveolin3 (23kDa) was observed in primary (A) and immortalized (IM) (B) cell lines after differentiation into myotubes. Mutations in dysferlin result in a reduced expression (IM DYSF1 and DYSF2/IM DYSF2) or in a complete absence of dysferlin protein (230kDa) (DYSF3/IM DYSF3 and DYSF4/IM DYSF4) in both myoblasts and myotubes. a-tubulin (50kDa) has been used as a loading control.  

In primary and immortalized wild-type myotubes and in IM DYSF1 and DYSF2/IM DYSF2 myotubes dysferlin was only expressed as a single 230 kDa protein band as revealed by the HAMLET antibody directed against the C-terminal juxtamembrane region of dysferlin and by a polyclonal antibody to the N-terminal part of dysferlin [10] (data not shown). This suggests that dysferlin protein with disease causing-point mutations is expressed as the full-length protein.

Altogether dysferlin-deficient immortalized human myoblasts showed a differentiation pattern comparable to their untransformed counterparts. Furthermore, the differentiation of myoblasts with disease-causing mutations in dysferlin into myotubes is similar to wild-type myoblasts with respect to the expression of myogenic differentiation markers such as desmin, MHC and caveolin3. 

### Differentiation pattern of dysferlin-deficient and wild-type immortalized human myoblasts 

Myogenic differentiation of dysferlin-deficient and wild-type immortalized human myoblasts into myotubes was followed by immunocytochemistry to reveal the expression of early and late myogenic differentiation markers including the myogenic regulatory transcription factors MyoD and myogenin, muscle structural proteins MHC, a-actinin and desmin and differentiation-regulated sarcolemmal proteins dysferlin and caveolin3. Representative examples are shown in Figure 2. After 7 days of differentiation multinucleated myotubes were formed from dysferlin-deficient and from wild-type immortalized human myoblasts as demonstrated by the expression of MHC, a-actinin and caveolin3 (Fig.2). Dysferlin was present in wild-type myotubes, showed a reduced expression in IM DYSF1 and IM DYSF2 and was completely absent in IM DYSF3 and IM DYSF4 (Fig.2). 

**Figure:**
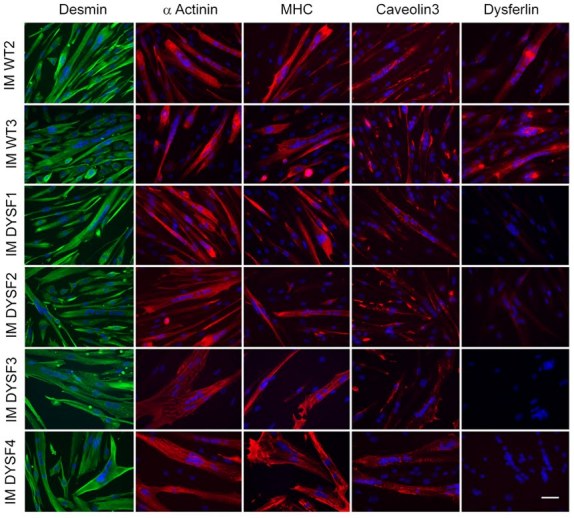
**Fig.2** Differentiation state of myotubes derived from human immortalized dysferlin-deficient and wild-type myoblasts. Immunofluorescence stainings show the formation of multinucleated myotubes expressing the myogenic differentiation markers desmin, a-actinin, MHC and caveolin3 in both wild-type and dysferlin-deficient myotubes. Disease-causing mutations in dysferlin result in a reduced expression (IMDYSF1 and IM DYSF2) or in a complete absence of dysferlin (IM DYSF3 and IM DYSF4). (bar, 50µm)

The subcellular localization of muscle differentiation-associated proteins was analyzed by high-resolution laser scanning confocal microscopy. After 7 days of differentiation a striated staining pattern for MHC and a-actinin, labelling Z-lines, was observed in myotubes derived from wild-type and dysferlin-deficient immortalized myoblasts (Fig.3). This is indicative of the correct formation of sarcomeres in these myotubes independent of the presence or absence of dysferlin. Accordingly, spontaneous contractions of myotubes were occasionally observed.

In wild-type myotubes dysferlin was distributed in the plasma membrane and the reticulate structures throughout the cell. Myotubes with disease-causing mutations in dysferlin showed an intracellular aggregation (IM DYSF1 and IM DYSF2) or a complete absence of dysferlin protein (IM DYSF3 and IM DYSF4) (Fig.3). 

**Figure:**
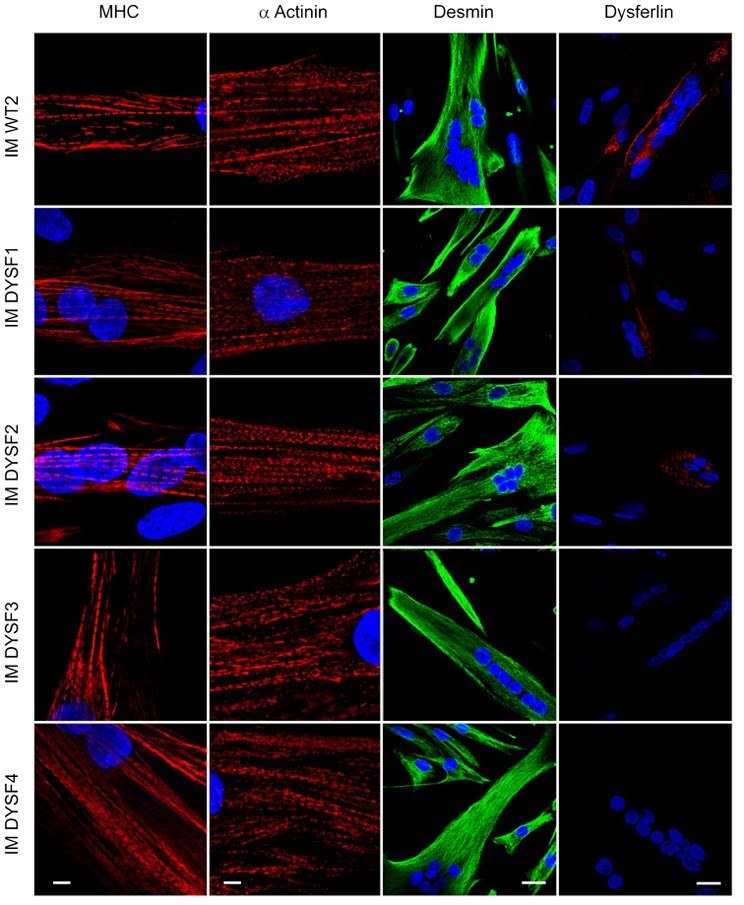
**Fig.3** ****Subcellular localization of muscle differentiation-specific proteins in myotubes derived from human immortalized dysferlin-deficient and wild-type clones of myoblasts.** **Immunofluorescence staining with either anti-MHC or with anti-α-actinin antibody revealed correct organization of the sarcomere and Z-lines, respectively, in all cell lines. Dysferlin was distributed to the plasma membrane in wild-type immortalized myotubes (IM WT2). Disease-causing mutations in dysferlin result in an intracellular aggregation (IM DYSF1 and IM DYSF2) or a complete absence of dysferlin protein (IM DYSF3 and IM DYSF4). (bar 5µm, left panels; bar 20µm, right panels)

### Dysferlin-deficiency and membrane repair

Since dysferlin is involved in plasma membrane resealing after injury, laser wounding assays were performed. Myotubes derived from dysferlin-deficient and wild-type immortalized human myoblasts were compared with respect to membrane repair. Membrane wounding was conducted employing a laser confocal microscope and the fluorescent membrane dye FM1-43 was used as a readout of membrane repair, as described by Cai et al. [14]. In myotubes derived from dysferlin-deficient immortalized myoblasts, we observed an opening of the targeted membrane frontier and a staining of intracellular membrane compartments by the influx of the fluorescent dye FM1-43 (Fig.4B and 4C). Membrane integrity was not restored during a 280 sec observation period. In myotubes from wild-type immortalized human myoblasts, we observed resealing of the targeted membrane frontier after laser wounding and no influx of FM1-43 during the remaining 280 sec (Fig.4A). These observations were reinforced by the quantification of the increase in fluorescence intensity in dysferlin-deficient compared to wild-type myotubes (Fig.4D). 

**Figure:**
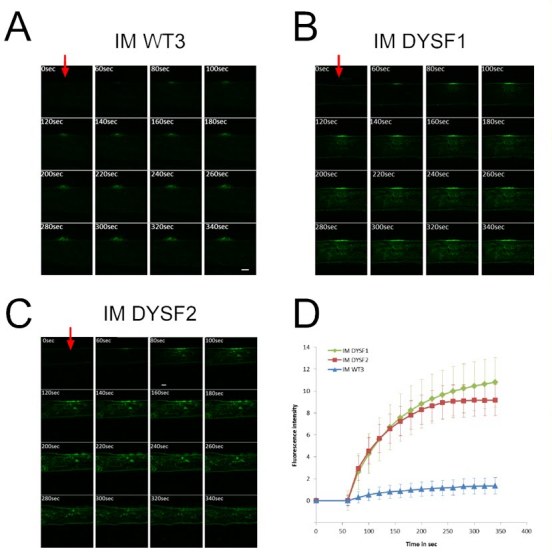
**Fig.4 **Laser mediated membrane wounding as an assay for dysferlin protein function.Myotubes derived from immortalized human myoblasts were wounded by laser irradiation and FM1-43 influx was visualized every 20 sec. Dysferlin-deficient myotubes (B and C; IM DYSF1 and IM DYSF2) show an influx of FM1-43 after membrane wounding that was not observed in wild-type myotubes (A; IM WT3). Summary data for FM1-43 dye entry into myotubes following laser mediated membrane wounding are shown in (D). Data represent means ±SE (n=7, IM WT3; n= 8, IM DYSF1; n=11, IM DYSF2). (bar, 5µm)

## Discussion 

Primary human myoblasts are an indispensible tool to study cellular processes in muscle disease. However, the material is very precious and available in very small quantities because of the limited size of biopsy procedures that can be performed and the reduced proliferative potential of human myoblasts which will be further reduced in muscular dystrophies due to the cycles of degeneration and regeneration. Furthermore, primary cultures from dystrophic muscle are generally highly intermingled with fibroblasts that cannot always be sorted completely. In the past, we observed that cultures derived from different LGMD2B patients consisted mainly of fibroblasts and adipocytes and we were therefore unable to generate pure primary myoblast lines from them, as examplified for DYSF1 in this report. However, in the present study it was possible to get a 100% myogenic population of IM DYSF1 after immortalization with hTERT and CDK-4 and subsequent cloning highlighting the great potential of the immortalization procedure. We thoroughly characterized four immortalized LGMD2B patient cell lines harbouring different dysferlin mutations not only for myogenic differentiation but also functionally and found no significant differences to their parental cell lines (Fig.1 and data not shown). Therefore, the immortalization of primary human myoblasts represents a major advantage and may overcome the limitations mentioned above. In addition to LGMD2B, immortalized human myoblast lines have been successfully established from patients with other muscle diseases including facioscapulohumeral MD, Duchenne MD, congenital MD and oculo-pharyngeal MD [13] [15].  

Human cell lines with unlimited proliferative capacity are useful tools in cell biology and for translational research. Up to now, only murine myoblast lines are available that are deficient for dysferlin. GREG cells were derived from A/J mice lacking dysferlin expression [16] and a C2C12 cell clone with a stable dysferlin knock-down by shRNA has been establihed that expresses about 10% of residual dysferlin [17]. 

There are discrepancies in the literature about the potential of dysferlin-deficient myoblasts to fuse and to differentiate into myotubes. Dysferlin-deficient myoblasts have been described to fully differentiate without any impairment as compared to wild-type myoblasts with respect to myoblast fusion and activation of myogenic pathways. This strongly suggests that dysferlin does not play a role in early stages of myotube formation and subsequent maturation [7] [18] [19] [20] [21]. Instead, myoferlin, another member of the ferlin protein family, has been described to be essential for myoblast-myoblast and myoblast-myotube fusion [18] [22]. Myoferlin is expressed early during myoblast differentiation whereas dysferlin is expressed only after the formation of multinucleated myotubes [18] [22]. Contradictorily, a reduced or delayed differentiation of dysferlin-deficient myoblasts into myotubes has been attributed to dysferlin-deficiency by other groups [17] [23] [24]. Reasons for these discrepancies might be caused by the use of different cellular models, species, culture conditions, *in vitro* cultivation time, experimental design or developmental differences (e.g. age of the donor patient) that finally result in a different myogenic potential and differentiation kinetics. For instance it has been shown that differentiation kinetics of immortalized myoblast lines slow down with time in culture probably due to constant selection for proliferation [15]. 

Being able to assess new therapeutical approaches is of great significance and requires to prove the proper function of the restored protein. This can be achieved partially by analysis of the correct intracellular localisation and the size of the protein using immunochemical approaches. In the case of dysferlin the assumption that dysferlin is indispensable in sarcolemmal repair opens the possibility for a direct functional assay by laser-mediated membrane wounding in cultured myotubes and myofibers. We show here that myotubes derived from the immortalized dysferlin-deficient myoblast lines, e.g. IM DYSF1 and IM DYSF2, can be employed as a read-out tool of dysferlin functionality by laser-mediated wounding of the sarcolemma. Our results are in accordance with the earlier observed dysfunction of the membrane resealing process in the absence of dysferlin in myotubes and myofibers [6] [8] [14]. We conclude that the human immortalized dysferlin-deficient myoblast lines represent innovative tools to assess dysferlin functionality after application of pharmacological and genetical approaches to restore dysferlin.

Although we did not analyze cellular metabolism and regulation of cell cycle progression we expect metabolic changes in immortalized myoblasts due to their high proliferative potential. However, this seems to have no influence on myogenic differentiation and dysferlin function in immortalized myoblasts and their corresponding myotubes as demonstrated in this report. 

In summary, the immortalized myoblast cell lines display properties highly similar to their parental cell lines with respect to myogenic differentiation, formation of multinucleated myotubes, development of a correct myofibrillar architecture and dysferlin protein expression. Dysferlin reveals unaltered subcellular localization and function in membrane repair in control cell lines, while it is perturbed in cell lines derived from LGMD-2B patients. In addition dysferlin-deficient myoblasts have been described to fully differentiate suggesting that dysferlin does not play a role in early stages of myotube formation. Therefore, immortalized human myoblast lines harbouring different mutations in dysferlin represent a very useful tool to further investigate dysferlin function, to study the pathophysiological mechanisms involved in dysferlinopathy and more importantly to assess therapeutic strategies to correct dysferlinopathies with a reliable readout.

## Acknowledgements 

We thank Stephanie Meyer for expert technical assistance. 

## Funding information

The Deutsche Forschungsgemeinschaft funded the study through Myograd (GK1631) and the clinical research group KFO192 “Skeletal muscle growth regulation and dysregulation”. The Jain Foundation and the Association Française contre les Myopathies (AFM) also supported this investigation. 

## Competing interests

The authors have declared that no competing interests exist.

## Address for Correspondence

Simone Spuler, MD 

ECRC, Charité Campus Buch, Lindenberger Weg 80, 

13125 Berlin, Germany

T+49 30 450 540501,  F: +49 30 450 540906, e-mail: simone.spuler@charite.de
